# Comparative Effects of Cigarette Smoke and Heated Tobacco Product Aerosols on Biofilm Production by Respiratory Pathogens

**DOI:** 10.3390/microorganisms13112459

**Published:** 2025-10-28

**Authors:** Pavel Schiopu, Dan Alexandru Toc, Ioana Alina Colosi, Carmen Costache, Paul-Ștefan Panaitescu, Vlad Sever Neculicioiu, Codrina Mihaela Gorcea, Tudor-Ioan Zăgărin, Andreea Roxana Murarasu, Doina Adina Todea

**Affiliations:** 1 Department of Microbiology, “Iuliu Hațieganu” University of Medicine and Pharmacy, 400012 Cluj-Napoca, Romania; pavel.schiopu@umfcluj.ro (P.S.);; 2 Department of Pneumology, “Iuliu Hațieganu” University of Medicine and Pharmacy, 400332 Cluj-Napoca, Romania; dtodea@umfcluj.ro; 3 Faculty of Medicine, “Iuliu Hațieganu” University of Medicine and Pharmacy, 400012 Cluj-Napoca, Romania

**Keywords:** biofilm, heated tobacco products, tobacco heating products, cigarette smoke, *Staphylococcus aureus*, *Pseudomonas aeruginosa*, *Klebsiella pneumoniae*, *Streptococcus pneumoniae*, *Haemophilus influenzae*, NTHi, respiratory pathogens, COPD

## Abstract

Biofilms are involved in both acute and chronic respiratory infections. While cigarette smoke extract (CSE) has been shown to increase biofilm formation by certain respiratory pathogens, the impact of emerging heated tobacco products (HTPs) remains unclear. We compared the effects of CSE with two HTP aerosol extracts on biofilm biomass and metabolic activity of common respiratory pathogens. Reference strains of *Staphylococcus aureus*, *Pseudomonas aeruginosa*, *Klebsiella pneumoniae*, *Streptococcus pneumoniae*, and non-typeable *Haemophilus influenzae* (NTHi), known respiratory pathogens, were grown as 24 h biofilms in 96-well plates (48 h for *S. aureus* and *P. aeruginosa*). These were exposed to CSE and HTP extracts from iQOS™ (Terea™ Turquoise, ILUMA™ device) and glo™ (neo™ Azure, HyperPro™ device), prepared in liquid culture media. Biofilm density was quantified by the crystal violet assay. Metabolic activity (planktonic and biofilm) was assessed by MTT reduction to formazan. At 24 h, CSE markedly reduced *H. influenzae* biomass versus iQOS™, glo™, and control, while *K. pneumoniae*, *S. aureus*, and *P. aeruginosa* showed no significant biomass differences. At 48 h, CSE significantly increased biomass in *P. aeruginosa* and *S. aureus* versus other exposures. Biofilm MTT assay measured metabolic activity increased in CSE exposure for *K. pneumoniae* versus iQOS™ and control, and for *S. aureus* versus control. Overall, HTP extracts showed limited, inconsistent effects compared with CSE, indicating combustion-derived constituents more strongly promote biofilm maturation in this model.

## 1. Introduction

### 1.1. Biofilm Formation in the Pathogenesis of Acute and Chronic Lung Infections

Bacterial biofilms are structured communities of microorganisms encased in a self-produced extracellular matrix (“slime”) composed of polysaccharides, proteins, and extracellular DNA (eDNA). This matrix acts as a protective barrier, shielding bacteria from antibiotics and the host’s immune system, contributing to persistent infections and chronic inflammation [1].

Biofilms are implicated in respiratory infections, both acute and chronic. As early as 1980, the presence of *P. aeruginosa* mucoid microcolonies in cystic fibrosis pulmonary tissue was demonstrated [2]. They have been detected in the sputum of patients suffering from pneumonia and chronic obstructive pulmonary disease (COPD) [3,4,5], being described as similar to those in cystic fibrosis (CF) [3], where their role in pathogenesis is better established [6]. They can rapidly colonize endotracheal tubes and serve as polymicrobial reservoirs that seed ventilator-associated pneumonia [7].

Previously thought to be a means of bacterial persistence in chronic infections, biofilms have been shown to predominate versus planktonic bacteria in sputum samples not only in chronic conditions such as COPD and CF, but also in community-acquired pneumonia (CAP), an acute disease, using quantitative confocal fluorescence microscopy [5].

The clinical relevance of these findings comes from the fact that both bacterial and fungal biofilms can be extremely resistant to treatment or removal. In diseases of the respiratory system, such as CF and *Aspergillus* infections, drug-tolerant biofilm aggregates have been shown to drive chronic disease and treatment failure [7].

### 1.2. Smoking and COPD

Smoking remains one of the most significant risk factors for COPD [8]. Compared to non-smokers, cigarette smokers experience respiratory symptoms and impaired lung function more frequently, have a more rapid yearly decline in forced expiratory volume in 1 s (FEV_1_), and face a higher risk of dying from COPD [9]. Despite its known harm, around 40% of patients with COPD continue to smoke, which worsens symptoms and long-term outcomes [10,11].

Patients who continue to smoke have a significantly higher risk of hospitalization due to COPD exacerbations [12]. These can be triggered by viral, bacterial, or mixed respiratory infections, as well as by environmental factors such as air pollution or temperature changes. Bacterial or mixed (bacterial and viral) infections are implicated in nearly half (49.5%) of exacerbations [13].

Any effect of tobacco products on bacterial biofilm formation is therefore particularly relevant in COPD patients, since many continue smoking even after diagnosis, thereby exposing their respiratory microbiota to cigarette smoke, electronic cigarette vapor extract (ECVE), and heated tobacco product (HTP) aerosols.

### 1.3. Conventional Cigarette Smoke Effect on Biofilm Formation by COPD and Pneumonia Associated Pathogens

The most frequently isolated bacteria in COPD exacerbations are *Haemophilus influenzae*, *Pseudomonas aeruginosa*, *Acinetobacter baumannii*, *Klebsiella pneumoniae*, *Streptococcus pneumoniae*, *Escherichia coli*, *Haemophilus parainfluenzae*, and *Staphylococcus aureus* [14].

Among them, *S. aureus*, *P. aeruginosa*, and *S. pneumoniae* have been found to produce a denser biofilm when exposed to cigarette smoke extract/condensate [15,16,17,18,19,20,21,22].

### 1.4. Emergence of Heated Tobacco Products (HTPs) Such as iQOS™ and Glo™

In recent years, the tobacco industry has reintroduced heated tobacco products (HTPs), also called tobacco heating products. The main characteristic that differentiates HTPs from regular, traditional combustible cigarettes (TCCs) is that it is heated (using a specific electronic device) to a lower temperature, 350–550 °C, than when they are burned in conventional cigarettes (900 °C) [23,24]. However, charring due to pyrolysis has also been demonstrated in iQOS™ [23]. Innovative technologies, design, marketing, and health-related claims associated with these products have weakened tobacco control measures in some countries, where there were relatively strict laws for regulating conventional cigarettes, and attempts by the tobacco industry to reposition itself as a public health partner. Descriptions such as “heat-not-burn”, “smoke-free”, and “ash-free” have prompted regulatory disputes over inclusion in smoke-free policies. HTPs generate aerosols that contain toxic products, many at lower levels than those in conventional cigarette smoke, but in some cases at higher levels. These harmful and potentially harmful constituents (HPHC–Harmful and Potentially Harmful Constituents) include nicotine, carbon monoxide, formaldehyde, acrolein, and various tobacco-specific nitrosamines [25].

Although some studies show that these products generate fewer HPHCs overall compared to traditional cigarettes [26,27] many of those studies are funded by tobacco industry companies [27,28]. The WHO maintains that a lower level of HPHCs does not necessarily mean a lower risk and that the long-term health effects of these products remain unknown, and further studies should be conducted to determine health risks [25].

It is expected that HTPs will achieve a significant market share [29]. A meta-analysis including studies from 42 countries found a 1.5% prevalence of HTP use. A proportion of 5% of the population has used HTPs at least once, with a prevalence of 5.25% among adolescents. Usage is higher in Europe and the Western Pacific region, with an upward trend between 2015 and 2020 [30].

### 1.5. HTP Substitution in COPD

There is some interest regarding the health effects of substituting cigarettes with HTPs as a therapeutic intervention in COPD. One small cohort study has evaluated the long-term impact of HTP use in COPD patients, showing that switching from cigarettes to HTPs, either exclusively or with a ≥70% reduction in cigarette use, led to sustained improvements over three years. Patients experienced about a 40% reduction in exacerbations and respiratory symptoms, higher exercise tolerance, and improved quality of life, outcomes comparable to those seen with rehabilitation programs or standard medications [31]. However, the Global Initiative for Chronic Obstructive Lung Disease (GOLD) guidelines maintain that quitting smoking has the greatest potential to improve quality of life, reduce symptom burden, and lower the frequency of exacerbations [8], and do not currently recommend switching to heated tobacco products (HTPs). Similarly, a 2022 Cochrane review concluded that there is insufficient evidence to support recommending HTPs as a smoking cessation method [28].

We chose to investigate the tobacco products in equal concentrations in cigarettes or sticks per volume of media, to better reflect the practical exposure of a user. The number of cigarettes smoked versus the number of HTP sticks consumed per day does not differ by a large amount. Daily smokers smoke 16 cigarettes per day, while daily iQOS users consume on average 15 sticks per day, and glo users 13 sticks per day [32].

### 1.6. Gaps in Knowledge About the Effect of Cigarette Smoke and HTPs on Biofilm Formation

Cigarette smoke has been shown to increase biofilm formation by the respiratory tract pathogens, namely *S. pneumoniae*, *S. aureus*, and *P. aeruginosa* [15,16,17,18,19,20,21,22]. An effect on *H. influenzae* has been investigated but not demonstrated [22]. We have not been able to identify studies investigating this effect in *K. pneumoniae*, another important respiratory pathogen.

For HTPs, however, there is very little published evidence of biofilm promotion. Given that in some respiratory pathogens (*S. aureus*, *P. aeruginosa*, and *S. pneumoniae)* an increase in biofilm formation was demonstrated not only with CSE exposure but also with electronic cigarette vapor extract (ECVE) exposure [22], in which combustion compounds are absent, it is plausible that HTPs would have a similar effect.

Any biofilm-promoting activity of HTPs would be highly relevant to their evaluation as substitution therapies, given that biofilm formation is a well-recognized pathogenic mechanism in many infectious diseases, including respiratory infections [6,33], where it contributes to chronicity and renders antimicrobial therapy less effective [33].

### 1.7. Aim

The aim of this study was to investigate the effects of vapors generated by heated tobacco products (HTPs) on biofilm formation by bacteria frequently involved in respiratory infections, and to compare these effects with those of cigarette smoke.

The hypothesis was that HTP vapors increase biofilm formation by these respiratory pathogens.

## 2. Materials and Methods

### 2.1. Bacterial Strains

Overnight cultures of bacterial strains were performed from frozen (−80 °C) stocks on solid non-selective media, namely *S. aureus* ATCC 25923, *P. aeruginosa* ATCC 27853, *K. pneumoniae* ATCC 700603, *S. pneumoniae* ATCC 49619, on Columbia agar with sheep blood (Oxoid, Basingstoke, UK), and non-typeable *H. influenzae* ATCC 49247 on chocolate agar (Bio-Rad, Hercules, CA, USA).

### 2.2. Preparation of Smoke and Vapor Extracts

In order to expose the bacteria to the various water-soluble compounds produced by cigarettes and HTPs, an established method was adapted [34]. Smoke from Marlboro Red™ cigarettes (containing 0.8 mg nicotine, 10 mg tar, and 10 mg carbon monoxide per cigarette) was bubbled through liquid culture media. The HTPs used were Terea™ (Philip Morris International Inc., Stamford, CT, USA) turquoise variant (4/10 intensity listed on the manufacturer’s website), used with an iQOS™ ILUMA™ device and glo™ neo™ Azure sticks with a glo™ HyperPro™ device, used in boost mode, according to the manufacturer’s instructions.

The concentrations used were 2 cigarettes per 50 mL and 2 HTP sticks per 50 mL. To approximate real-world exposure, we normalized extract concentrations by unit of product consumed—one conventional cigarette or one heated tobacco product (HTP) stick—per volume of medium. This approach is behavior-anchored, as daily consumption is broadly similar across products; daily cigarette smokers average ~16 cigarettes/day, while daily HTP users report comparable stick counts (IQOS ≈ 15 sticks/day; glo ≈ 13 sticks/day) [32].

The media utilized were BHI (Brain Heart Infusion) broth (Oxoid, United Kingdom), sBHI–BHI supplemented with 10 µg/mL hemin (Oxoid, United Kingdom), and 10 µg/mL β-NAD (Oxoid, UK), and TSB–Tryptone Soy Broth (Oxoid, UK). For *S. aureus*, *P. aeruginosa*, and *K. pneumoniae*, the extracts were prepared in TSB; for *S. pneumoniae* and non-typeable *H. influenzae*, the extracts were prepared in BHI and sBHI.

The resulting liquids were filter sterilised using 0.45 μm and 0.2 μm filters (Macherey-Nagel, Hoerdt, France).

### 2.3. Evaluating Biofilm Density: Biofilm Culture and Crystal Violet Staining

Biofilms were grown in tissue-culture-treated 96-well polystyrene plates (VWR, Radnor, PA, USA) according to a published protocol [35]. Bacterial suspensions were prepared by adjusting overnight liquid cultures to 1.5 × 10^8^ CFU/mL using a McFarland densitometer (BioMérieux, France), then diluting 100 μL into 10 mL of species-appropriate broth. The target concentration was 7.5 × 10^5^ CFU/mL (final in well) and was verified by serially diluting and plating on solid media.

Wells were inoculated with 100 μL bacterial suspension and 100 μL of smoke/vapor extract. The microtiter plates were incubated for 24 h at 37 °C, with 10% CO_2_ for *S. pneumoniae* and *H. influenzae*. A water tray was placed inside the incubator to maintain high humidity. For *S. aureus* and *P. aeruginosa* 48 h biofilms were grown as well, with media replenishment (1/4 of volume) after 24 h. After removing the planktonic cells, biofilms were fixed with methanol (20 min), stained with 0.1% crystal violet *w*/*v*, eluted in 95% ethanol, and absorbance was measured at 620 nm using a microplate spectrophotometer type 250 (BioMérieux, Craponne, France).

On each plate, six wells were filled only with culture media (negative controls), and three wells were assigned to each species + exposure condition. The whole experiment was repeated three times.

Following incubation, planktonic cell optical density at 620 nm was measured as well to determine if the extracts had bacteriostatic effects. The media used as a negative control was inoculated on Columbia agar to verify sterility. The content of wells containing only bacteria (growth controls) was plated as well to check culture purity.

### 2.4. Evaluating Metabolic Activity of Planktonic Bacteria

The metabolic activity of CSE and HTP-exposed planktonic cultures was measured in a 3-(4,5-dimethylthiazol-2-yl)-2,5-diphenyltetrazolium bromide (MTT) assay [36]. Briefly, after 18 h of incubation, the supernatant containing planktonic cells was carefully transferred to new sterile plates to avoid disturbing the adherent biofilms. A freshly prepared MTT solution (final concentration 0.5 mg/mL) was added, and plates were incubated for 4 h at 37 °C, protected from light. Metabolically active bacteria reduced MTT to purple formazan crystals, which were subsequently solubilized using 100 μL dimethyl sulfoxide (DMSO). After a 20 min incubation on an orbital shaker (70 RPM), absorbance was measured at 620 nm using 690 nm as a reference wavelength. Background absorbance from blank wells was subtracted, and results were normalized to negative control wells (liquid culture media). The reagents were procured from Sigma-Aldrich (Sigma-Aldrich, St. Louis, MO, U.S.A.)

### 2.5. Evaluating Biofilm Metabolic Activity

After 18 h of incubation, planktonic cells and spent medium were carefully removed, and wells were washed three times with sterile PBS to eliminate unattached bacteria. A freshly prepared MTT solution (0.5 mg/mL) was added to each well, and plates were incubated in the dark at 37 °C for 4 h to allow metabolically active cells within the biofilm to reduce MTT to purple formazan crystals. The MTT solution was then removed without disturbing the biofilm, and formazan crystals were solubilized by adding DMSO and shaking for 20 min at room temperature. Absorbance was measured at 620 nm using a microplate reader, with 690 nm as the reference wavelength. Background values from negative control wells (liquid culture media) were subtracted.

### 2.6. Fluorescent Staining and Microscopy

Biofilms were grown on steam-sterilized round glass coverslips placed in 6-well plates. Each well contained 1 mL of an overnight culture of the respective species, mixed 1:1 with the extracts described above. Uninoculated medium served as the negative control. Plates were incubated for 24 h at 37 °C. Following incubation, coverslips were rinsed twice with PBS, fixed in 4% paraformaldehyde, and blocked for 20–30 min with blocking buffer (10% sheep serum, 0.05% Tween-20 in PBS), then rinsed to minimize background.

Staining protocols varied by species. For *H. influenzae* biofilms, 200 µL of FilmTracer™ SYPRO^®^ Ruby Biofilm Matrix Stain (ready-to-use) was applied for 30 min at room temperature, followed by three rinses with filter-sterilized water.

*S. aureus* biofilms were first stained with 200 µL SYTO^®^ 9 (5 μM in PBS) for 15 min at room temperature in the dark. To visualize GlcNAc-rich exopolysaccharides and peptidoglycan, Alexa Fluor™ 594 wheat germ agglutinin (WGA; 5 μg/mL) was then applied for 20 min.

For *P. aeruginosa*, *K. pneumoniae*, and *S. pneumoniae*, the first stain was SYTO^®^ 9 as described above, followed by Texas Red^®^-conjugated concanavalin A (ConA; 200 µg/mL in PBS, 20 min) to detect biofilm matrix polysaccharides.

Between staining steps, coverslips were gently rinsed three times with PBS to reduce background and preserve biofilm structure. After a final rinse (twice with PBS, once with filter-sterilized water), coverslips were air-dried and mounted face-down on glass slides with a drop of anti-fade mounting oil.

The slides were examined using a Zeiss Axio Lab A1 epifluorescence microscope (Carl Zeiss Microscopy GmbH, Oberkochen, Germany), equipped with a 100×/1.25 oil immersion objective lens, a 470 nm LED with filter set 9, a 530 nm LED with filter set 14, and an Erc 5 s camera (Carl Zeiss Microscopy GmbH, Germany). Image post-processing consisted of a minimal contrast increase that was applied identically across all images within the same staining set.

All stains were obtained from Thermo Fisher Scientific, Waltham, MA, USA.

### 2.7. Statistical Analysis

For CV assays, 620 nm readings were first adjusted by subtracting ODc (the average OD of the negative control + 3 SD of the negative control), as recommended by Stepanović et al. [35]. For MTT assays, 620 nm was first adjusted by subtracting 690 nm as the reference wavelength, then subtracting the average OD of the negative (sterility) control wells containing only the liquid culture media.

The microtiter plate layout included three replicates per bacterium and condition, and the experiment was repeated three times.

A Kruskal–Wallis test within each strain–time subset to check for overall differences among groups without assuming normality. To account for multiple testing across species, the Benjamini–Hochberg procedure was applied to control the false discovery rate (FDR). The value of *p* < 0.05 was considered significant. Where the adjusted Kruskal–Wallis *p*-value was below 0.05, a Dunn’s post-hoc pairwise test with Holm correction was performed. Kruskal–Wallis significance levels are shown as text labels, while Dunn’s pairwise comparisons are marked with brackets and stars (“*” for *p* < 0.05, “**” for *p* < 0.01).

Box plots display the median as the central line, the box spanning the interquartile range (Q1–Q3), whiskers extending to the most extreme values within 1.5 × IQR, and individual points beyond the whiskers plotted as outliers.

The software used for running the statistical tests and graphics generation was R version 4.5.0 [37] using RStudio version 2025.05.01+513 [38].

## 3. Results

### 3.1. Effect of CSE and HTP Extract on Biofilm Density (Crystal Violet Assay)

**Figure 1:**
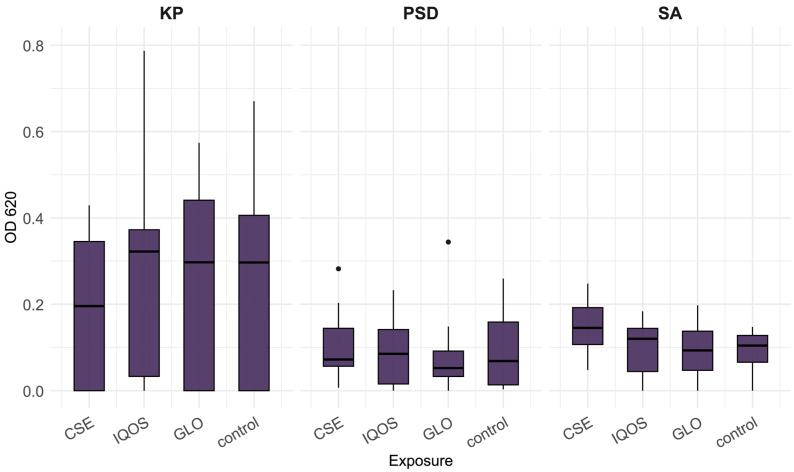
Optical density of *Klebsiella pneumoniae* (KP), *Pseudomonas aeruginosa* (PSD), and *Staphylococcus aureus* (SA) 24 h biofilms, following crystal violet staining, relative to tobacco product extract exposure; CSE = cigarette smoke extract, iQOS = iQOS vapor extract, GLO = glo vapor extract, control = unexposed bacteria.

**Figure 2:**
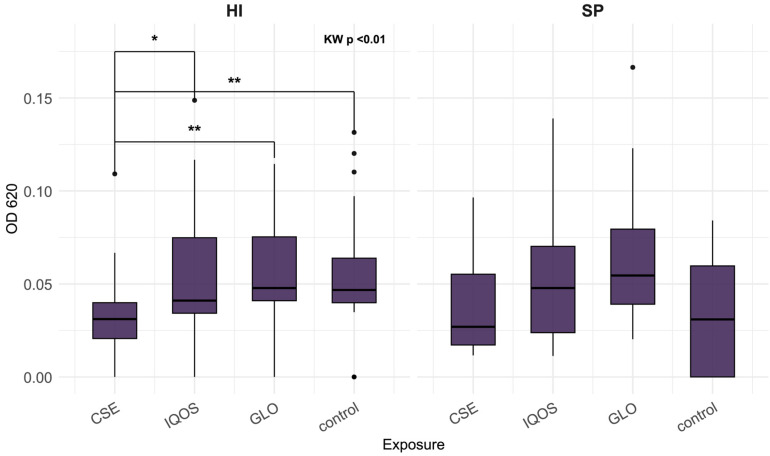
Optical density of *Haemophilus influenzae* (HI) and *Streptococcus pneumoniae* (SP) 24 h biofilms, following crystal violet staining, relative to tobacco product extract exposure; CSE = cigarette smoke extract, iQOS = iQOS vapor extract, GLO = glo vapor extract, control = unexposed bacteria. KW = Kruskal–Wallis H test. Significant Dunn’s pairwise comparisons are marked with brackets and stars (“*” for *p* < 0.05, “**” for *p* < 0.01).

**Figure 3:**
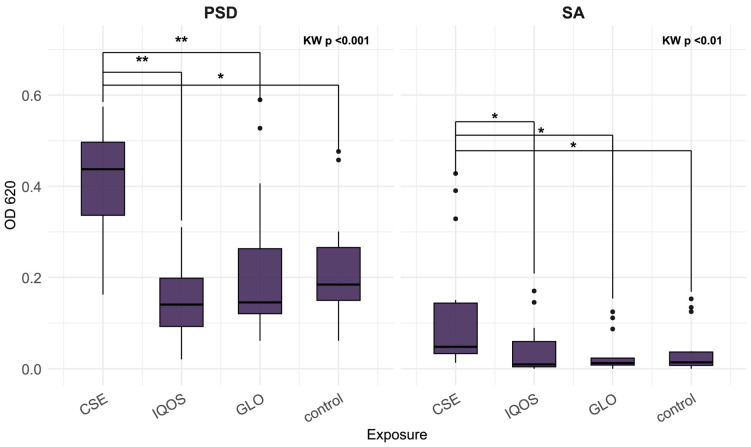
Optical density of *P. aeruginosa* (PSD) and *S. aureus* (SA) 48 h biofilms, following crystal violet staining, relative to tobacco product extract exposure; CSE = cigarette smoke extract, iQOS = iQOS vapor extract, GLO = glo vapor extract, control = unexposed bacteria. KW = Kruskal–Wallis H test. Significant Dunn’s pairwise comparisons are marked with brackets and stars (“*” for *p* < 0.05, “**” for *p* < 0.01).

The distributions for CSE, iQOS, glo, and control indicate that the effects of tobacco product extracts on 24 h biofilm vary across species, both in direction and magnitude (Figure 1, Figure 2 and Figure 3). At 24 h, *K. pneumoniae*, *S. aureus*, and *P. aeruginosa* biofilms did not differ significantly across exposures (Figure 1). In contrast, *H. influenzae* biofilms showed a marked reduction in biomass under CSE exposure compared with all other conditions, including the heated tobacco product (HTP) extracts (Kruskal–Wallis *p* < 0.01; Figure 2). By 48 h, the pattern shifted, CSE exposure resulted in substantially increased biofilm density in both *P. aeruginosa* and *S. aureus*, with median values for *P. aeruginosa* at least twofold higher than those observed for iQOS, glo, or control (Kruskal–Wallis *p* < 0.001 and *p* < 0.01, respectively; Figure 3). It is apparent that glo-exposed S. pneumoniae biofilms were the most dense; however, this was not a significant result (Figure 2).

### 3.2. Effect of CSE and HTP Extract on Bacterial Metabolic Activity (MTT Assay)

**Figure 4:**
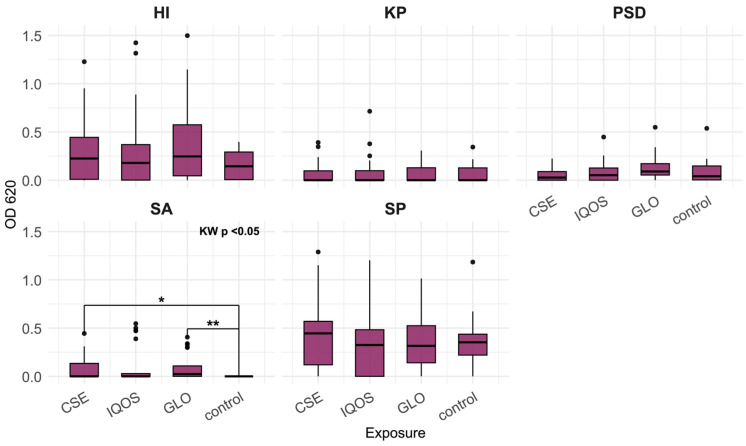
Planktonic cell metabolic activity measured by MTT assay (OD 620 nm–690 nm) of *H. influenzae* (HI), *K. pneumoniae* (KP), *P. aeruginosa* (PSD), *S. aureus* (SA), and *S. pneumoniae* (SP) after 24 h of incubation, relative to tobacco product extract exposure; CSE = cigarette smoke extract, iQOS = iQOS vapor extract, GLO = glo vapor extract, control = unexposed bacteria. KW = Kruskal–Wallis H test. Significant Dunn’s pairwise comparisons are marked with brackets and stars (“*” for *p* < 0.05, “**” for *p* < 0.01).

The results in Figure 4 show that the metabolic activity of *S. aureus* planktonic cells is significantly increased in both glo and CSE (*p* < 0.05).

### 3.3. Effect of CSE and HTP Extract on Biofilm Metabolic Activity (Biofilm MTT Assay)

**Figure 5:**
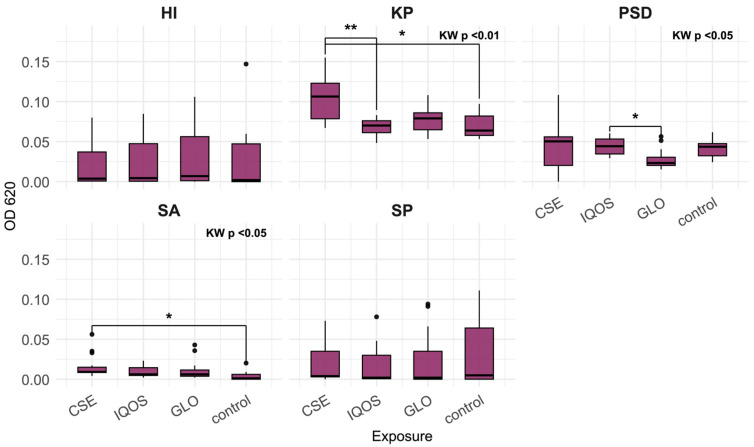
Biofilm metabolic activity measured by MTT assay (OD 620 nm–690 nm) of *H. influenzae* (HI), *K. pneumoniae* (KP), *P. aeruginosa* (PSD), *S. aureus* (SA), and *S. pneumoniae* (SP), after 24 h of incubation, relative to tobacco product extract exposure; CSE = cigarette smoke extract, iQOS = iQOS vapor extract, GLO = glo vapor extract, control = unexposed bacteria. KW = Kruskal–Wallis H test. Significant Dunn’s pairwise comparisons are marked with brackets and stars (“*” for *p* < 0.05, “**” for *p* < 0.01).

Figure 5 shows a more intense reduction of MTT to formazan in CSE-exposed biofilms relative to iQOS and control in the case of *K. pneumoniae* and relative to control in the case of *S. aureus*.

### 3.4. Biofilm Structure and Matrix Composition

**Figure 6:**
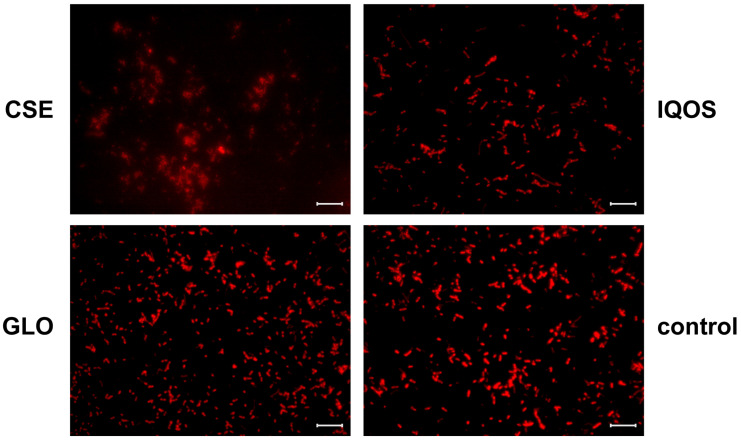
Epifluorescence microscopy images of 24-h *H.influenzae* biofilms, stained with FilmTracer SYPRO^®^ Ruby Biofilm Matrix Stain. The biofilms were exposed to: CSE, cigarette smoke extract, iQOS, iQOS vapor extract, GLO, glo vapor extract, and control, unexposed bacteria. Scale bar = 10 μm.

CSE exposure resulted in less dense *H. influenzae* biofilms than those formed under iQOS, GLO, or control conditions (Figure 6).

**Figure 7:**
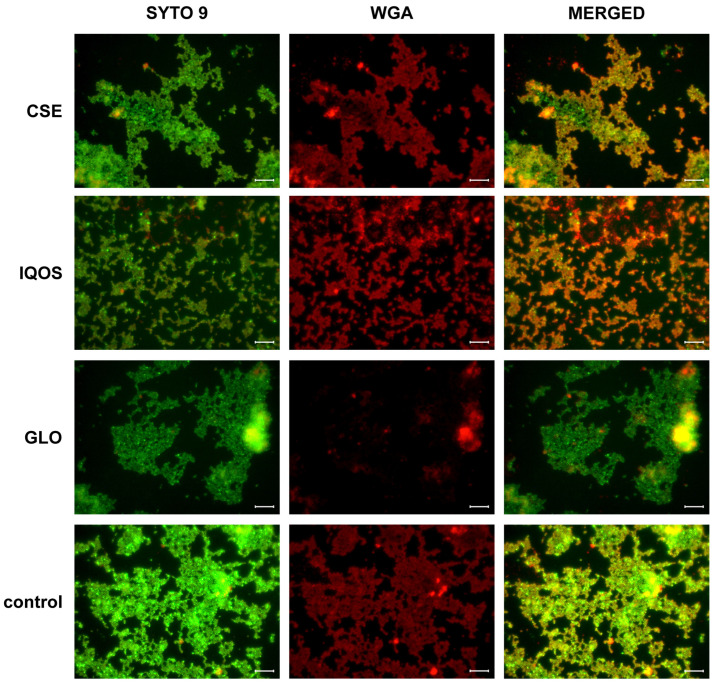
Epifluorescence microscopy images of 24-h *S. aureus* biofilms. Nucleic acids were stained with SYTO^®^ 9 (green), and peptidoglycan/polysaccharides were stained with wheat germ agglutinin conjugated to Alexa Fluor 594 (WGA, red). The biofilms were exposed to: CSE, cigarette smoke extract, iQOS, iQOS vapor extract, GLO, glo vapor extract, and control, unexposed bacteria. Scale bar = 10 μm.

In *S. aureus* biofilms, SYTO^®^ 9 and WGA staining show that glo exposure produced a decrease in WGA-AF594 signal, suggesting reduced GlcNAc-rich matrix (Figure 7).

**Figure 8:**
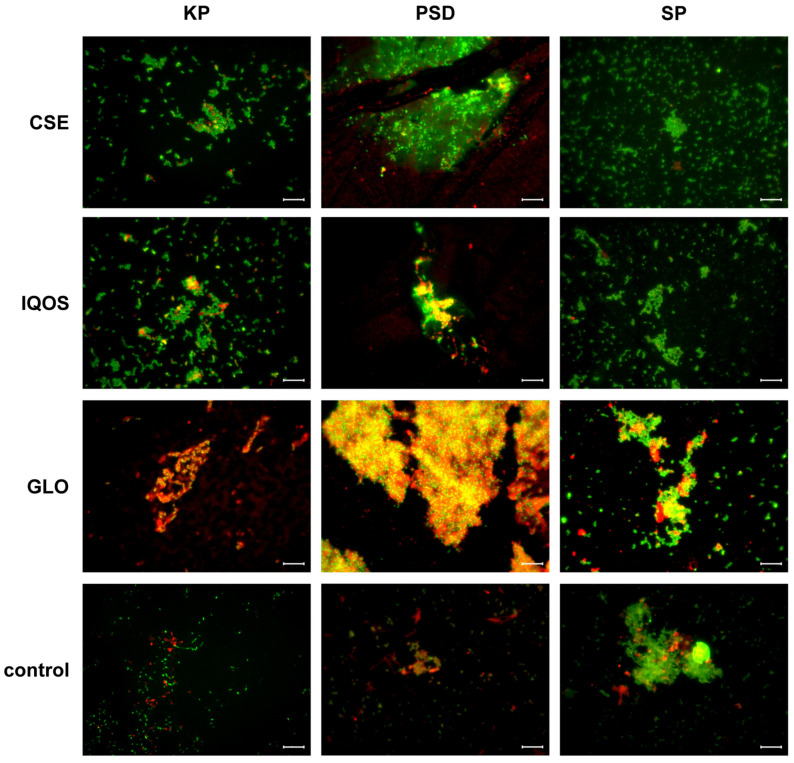
Merged epifluorescence microscopy images of 24-h *K. pneumoniae, P.aeruginosa*, and *S. pneumoniae* biofilms. Nucleic acids were stained with SYTO^®^ 9 (green), and polysaccharides were stained with concanavalin A conjugated to Texas Red^®^ (ConA, red). Exposure categories are: CSE, cigarette smoke extract; iQOS, iQOS vapor extract; GLO, glo vapor extract; and control, unexposed bacteria. Scale bar = 10 μm. Full panels of single-channel and merged images are available in Figures S1–S3.

Qualitative comparison of the micrographs suggests that biofilms exposed to glo extract exhibited stronger Texas Red-ConA staining of the extracellular matrix (Figure 8 and Figures S1–S3). This observation indicates a possible increase in matrix polysaccharides in *K. pneumoniae*, *P. aeruginosa*, and *S. pneumoniae* following glo exposure.

## 4. Discussion

Our study shows that, while tobacco products can increase biofilm production by species commonly isolated in respiratory infections, this effect is variable, depending on the type of tobacco product, the length of exposure, and the species/strain examined. This variability is a common theme across multiple studies in this area [39].

### 4.1. Biofilm Density

In *H. influenzae*, our crystal violet assay data show that CSE exposure produced a decrease in biofilm density compared to iQOS, glo, or control. One study investigated *H. influenzae* in vitro, finding no significant difference for CSE exposure and electronic cigarette vapor extract (ECVE) exposure [22]. By contrast, in an in vivo COPD ferret model, cigarette smoke exposure enhanced *H. influenzae* biofilm formation [40].

We did not find a significant effect in the case of *K. pneumoniae* biofilm density. One small study on chronic rhinosinusitis isolates has found an increase in biofilm density after, albeit with a slightly different method-direct colony exposure to tobacco smoke [16].

Both CSE and electronic cigarette vapor extract (ECVE) exposure have been shown to promote *P. aeruginosa* biofilm production [22]. We also observed an effect of CSE, but only in 48 h biofilms, and not 24 h biofilms. A study by Antunes et al. found that for *P. aeruginosa* in a slightly different 96-well plate model, found that 2 or 3 daily exposures were required for biofilm mass to increase significantly [21]. Smoke exposure was found to upregulate motility genes (*flgK*, *pilF*), which promote surface attachment and microcolony formation, and to alter quorum-sensing genes; *lasI* was upregulated, *rhlA* downregulated, and *lasB* unchanged. The extracellular matrix gene *algC* (involved in alginate biosynthesis) was also significantly induced. Parallel experiments with hydrogen peroxide showed that oxidative stress alone can trigger similar, dose- and time-dependent increases in biofilm mass, suggesting that smoke-induced oxidative stress is one driver of the phenotype [21].

An effect of CSE [19,22,41] and of ECVE [22] increases in the density of *S. aureus* biofilms were previously demonstrated. We replicated the effect of CSE on *S. aureus*, but only in more mature 48 h biofilms. This difference might highlight regional variation in the composition of unstandardized cigarettes, non-chemically defined culture media, or the polystyrene plates used. Kulkarni et al. have described a mechanism for this effect, namely the induction of oxidative stress, which upregulates oxidoreductase defenses, suppresses the *agr* quorum-sensing system, and stimulates adhesin expression. The resulting biofilms are rich in eDNA and proteins, more adhesive to host substrates, and likely more resilient [19]. One study found that nicotine exposure increased in *Staphylococcus aureus* biofilm formation in a dose-dependent manner, characterized by enhanced initial attachment, greater extracellular DNA release, and higher autolysis, with upregulation of *lytN* and *atlA*. These effects were linked to suppression of the accessory gene regulator (Agr) quorum-sensing system, since nicotine downregulated *agrA–D* and the phenotype was absent in *agrA* and *agrC* mutants [42].

Unlike prior reports, we did not observe a CSE-mediated increase in *S. pneumoniae* biofilm biomass [22]. Mutepe et al. showed denser pneumococcal biofilms in the presence of cigarette smoke, most notably at lower CSC concentrations, and they cautioned that prolonged, higher-dose CSC may inhibit growth [18]. Methodological and chemical differences likely explain the discrepancy with our following results: CSC (tar-rich, DMSO-solubilized) versus our aqueous CSE capture distinct smoke fractions, doses, and exposure kinetics; therefore, they can differ. In another study, across serotypes 6A, 19F, and 23F, CSC has repeatedly been linked to biofilm augmentation with early induction of TCS11 and stress/cation efflux genes (*cat eff*, *abc*), suggesting a conserved stress-response–linked mechanism with strain-dependent magnitude [17,43]. Complementing these findings, Manna et al. reported that CSE exposure of *S. pneumoniae* elicits a survival-oriented transcriptomic signature, upregulating TCS11, detox enzymes, efflux pumps, and competence genes, while downregulating pneumolysin and fatty-acid/D-alanylation pathways—without immediate changes in viability, adherence, hydrophobicity, or lysis susceptibility [44].

A similar CSC-induced biofilm density increase was also found to occur through oxidative stress–related mechanisms in *M. tuberculosis* [45].

### 4.2. Biofilm Metabolic Activity

The MTT assay quantifies NAD(P)H-dependent oxidoreductase activity in living bacteria, which reduces yellow MTT to insoluble purple formazan; the resulting absorbance is a proxy for cellular metabolic activity/viability. It has been previously used to test the metabolically inhibiting effect of antibiofilm agents in several studies [46,47,48].

We found that, in *Klebsiella pneumoniae*, biofilm metabolic activity was significantly increased in CSE, even though biomass did not differ significantly across conditions at 24 h, indicating early metabolic activation in the absence of detectable biomass augmentation.

In *Staphylococcus aureus*, CSE significantly enhanced metabolic activity at 24 h and an increase in biomass at 48 h, suggesting metabolic activation preceding biomass accumulation.

To our knowledge, there are no other studies measuring MTT reduction to formazan in the context of tobacco product exposure.

### 4.3. Growth and Metabolic Activity of Planktonic Cultures

We found that the metabolic activity of planktonic *S. aureus* cells was significantly increased in both glo and CSE. No significant decreases relative to control were found in this study.

Gilpin et al., using transmission electron microscopy and total viable count, found both CSE and ECVE to have no effect on the growth of any isolate tested [22]. This is consistent with our findings. One other study published only in abstract shows that the growth of planktonic cultures of *P. aeruginosa*, *S. pneumoniae*, and *H. influenzae* was not significantly inhibited by concentrations of CSE similar to those used in this study [49].

One study investigating the effect of CSE prepared with 6 cigarettes per 40 mL Mueller–Hinton broth and an extract of Heets sticks in an iQOS Duo device of 10 sticks per 40 mL on *S. pneumoniae* ATCC 49619 and *K. pneumoniae* ATCC 700603 found that both bacteria had an MIC of 6.25% *v*/*v* and an MBC of 12.5% *v*/*v* for the iQOS extract and MICs and MBCs of 6.25% *v*/*v* for the cigarette extract [50]. Converting these results for comparison, the MIC values are 0.47 cig/50 mL and 0.78 sticks/50 mL, so slightly below the 1 cigarette and 1 stick per 50 mL we used (when taking into account the 1:1 dilution we performed when mixing cultures with extracts in the microtiter plates).

### 4.4. Exposure to Tobbaco Extracts Modulates Biofilm Matrix Composition

The fluorescence imaging results were largely consistent with the quantitative assays while also providing qualitative insight into biofilm architecture and matrix composition. In *H. influenzae*, CSE exposure produced thinner, less confluent biofilms than iQOS, glo, or control, in line with the crystal violet assay showing reduced 24 h biomass. In an in vivo COPD ferret model, however, cigarette smoke exposure promoted *H. influenzae* biofilm formation, with an increase in lectin staining of the extracellular matrix [40].

In *S. aureus*, dual staining with SYTO^®^ 9 and WGA revealed that CSE exposure yielded more extensive nucleic acid and peptidoglycan/polysaccharide signal, whereas biofilms exposed to glo appeared to show weaker WGA staining. This suggests reduced accumulation of GlcNAc-rich matrix components compared with other conditions, even though biomass was not significantly different at 24 h. These qualitative differences are consistent with the 48 h CV assay and MTT assay results, which indicated enhanced biofilm growth and metabolic activity with CSE but not with glo. One study has found that pure nicotine enhances *S. aureus* biofilms through stronger attachment and an increase in eDNA release, but without increasing PIA (poly-N-acetylglucosamine, a GlcNAc-rich polysaccharide) [42].

In *K. pneumoniae*, *P. aeruginosa*, and *S. pneumoniae*, glo-exposed biofilms displayed stronger ConA staining of extracellular polysaccharides, suggesting an increase in matrix production. Although this effect did not translate into significant biomass differences in the crystal violet assay, it indicates that glo may alter extracellular matrix composition rather than overall cell number.

Microscopy supports some species and exposure-specific differences: CSE disrupted *H. influenzae* but promoted *S. aureus* and *P. aeruginosa* maturation, while glo exposure appeared to reduce matrix-associated staining in *S. aureus* yet enhance extracellular polysaccharide production in several gram-negative species.

To our knowledge, no previous study has applied matrix-specific fluorescence staining to visualize in vitro biofilm architecture of the included species under cigarette smoke or HTP exposure, making this a novel contribution.

One study on an unrelated species, *S. gordonii* found nicotine to enhance matrix polysaccharide synthesis within the biofilm, though the boost in extracellular polysaccharides (EPS) was not statistically significant [51]. *S. mutans* was found to produce less EPS when exposed to cigarette smoke [52].

### 4.5. Explanations for the Reduced HTP Effect

To our knowledge, no other studies have examined the effects of HTP extracts on biofilm formation by respiratory pathogens. In our study, we did not find the tested HTP extracts to significantly increase or decrease biofilm density or metabolic activity, even in species where we (or others) found CSE or ECVE to have an effect. This finding was not expected, given that HTPs generate aerosols that contain many potentially stressful/mutagenic compounds, even though they are present at lower levels than those in conventional cigarette smoke [26,27]. Furthermore, even electronic cigarette extracts/condensates have been shown to increase biofilm formation by some of these bacterial species, suggesting that combustion is not necessary for the production of biofilm-promoting compounds [22].

When comparing cigarette smoke and HTP aerosol, several chemical components stand out as particularly relevant for influencing bacterial biofilm formation. Nicotine is only slightly higher in TCCs vs. HTPs (0.5–1.50 mg/cigarette vs. 0.7–2.1 mg/HTP stick). Reactive oxygen species (ROS), however, are 1.5–8 times higher in TCC smoke than in HTP aerosol [53]. Given that oxidative stress often triggers protective bacterial responses, including an increase in production of extracellular matrix components and stress-regulated adhesins, which can enhance biofilm formation (as discussed above) this is a likely explanation for the HTPs comparatively low effect on biofilm formation.

Cigarette smoke has also been found to be more mutagenic than iQOS [50] and glo vapor [54] using the Ames test, although mutagenicity is at most a secondary driver of biofilm formation.

Different tobacco products can influence biofilm formation indirectly, by modifying surface characteristics (tar adhesion to surfaces, thermal effect, surface dissolving action of various aromatic hydrocarbons). For example, in an in vitro denture-materials model inoculated with *Streptococcus mutans*, HTP vapor was more effective than conventional cigarette smoke in increasing biofilm CFU count. However, this is not directly comparable to our study–surfaces were exposed to whole smoke and not aqueous CSE, so that the effect can be attributed mainly to the particulate fraction of the smoke. The authors attributed the increase primarily to smoke-induced increases in surface roughness, in line with prior work linking roughness to bacterial adhesion [55].

The difference between the effect of smoke and the effect of HTPs was investigated on bacteria implicated in other diseases. Both cigarette smoke and HTP aerosols promote a dysbiotic shift in the subgingival biofilm, favoring colonization by periodontopathogens, although the effect of HTP use was less pronounced than that of conventional smoking [56].

### 4.6. Limitations

This study employed a static in vitro 96-well biofilm model, meaning that no host factors, immune response, or airway surface fluid components were taken into account. We maintain that this model was appropriate given the exploratory nature of the research conducted.

Only one type of cigarette and two HTPs were included. The substantial variety of tobacco products on the market precludes any generalizable conclusion. This is compounded by the frequent changes to the availability and format of the devices and sticks. For example, we cited a 2025 study using Heets sticks with an iQOS Duo device [50], but at the date of publication of our study, these are already marked as discontinued on the local (Romanian) website, with the suggestion that users should switch to Terea sticks and the newer iQOS Iluma device [57].

We used only one concentration of CSE and HTP extract. Future studies should employ a range of concentrations of HTP vapor.

Clinical isolates might react differently to the reference strains we used. Given the genetic and metabolic diversity of clinical strains, we considered standard strains appropriate for this exploratory study. For example, *K. pneumoniae* strains isolated from sputum have been found to form no biofilm [58], and to form moderate to strong biofilms [59] in different studies. Patient characteristics could influence biofilm formation as well: in a study of bacteria isolated from chronic rhinosinusitis patients, it was found to be more likely that smoke exposure would increase biofilm formation if said bacteria were isolated from smokers [16].

This study does not provide insight into which exact substances produced by cigarettes and HTPs are responsible for the microbiological effects. Cigarette smoke alone contains over 8400 chemical components that could plausibly have an effect and/or various synergisms/antagonisms [60], requiring further studies.

## 5. Conclusions

This study demonstrates that cigarette smoke extract (CSE) has a stronger and more consistent impact on biofilm formation and metabolic activity of respiratory pathogens than heated tobacco product (HTP) extracts. CSE reduced early biofilm biomass in *H. influenzae*, but an increase in biomass in mature *S. aureus* and *P. aeruginosa* biofilms and enhanced metabolic activity in early *S. aureus* and *K. pneumoniae* biofilms. By contrast, HTP extracts (iQOS and glo) showed little effect, with only glo-exposure associated with an increase in extracellular polysaccharide staining in some gram-negative species. These findings support the idea that combustion-derived constituents, particularly reactive oxygen species, play a key role in driving biofilm-promoting phenotypes. The absence of significant HTP effects in our model does not exclude potential impacts under different conditions. Future studies should investigate a broader range of concentrations, clinical isolates, and airway-mimetic models, while also identifying the chemical mediators of these effects.

## Data Availability

The datasets presented in this article are not readily available because the data are part of an ongoing study. Requests to access the datasets should be directed to the corresponding authors.

## Supplementary Materials

The following supporting information can be downloaded at: https://www.mdpi.com/article/10.3390/microorganisms13112459/s1, Figure S1: Epifluorescence microscopy images of 24-h *K. pneumoniae* biofilms. Nucleic acids were stained with SYTO^®^ 9 (green), and polysaccharides were stained with concanavalin A conjugated to Texas Red^®^ (ConA, red). Exposure categories are: CSE, cigarette smoke extract; IQOS, IQOS™ vapor extract; GLO, glo™ vapor extract; control, unexposed bacteria. Scale bar = 10 μm; Figure S2: Epifluorescence microscopy images of 24-h *P. aeruginosa* biofilms. Nucleic acids were stained with SYTO^®^ 9 (green), and polysaccharides were stained with concanavalin A conjugated to Texas Red^®^ (ConA, red). Exposure categories are: CSE, cigarette smoke extract; IQOS, IQOS™ vapor extract; GLO, glo™ vapor extract; control, unexposed bacteria. Scale bar = 10 μm; Figure S3: Epifluorescence microscopy images of 24-h *S. pneumoniae* biofilms. Nucleic acids were stained with SYTO^®^ 9 (green), and polysaccharides were stained with concanavalin A conjugated to Texas Red^®^ (ConA, red). Exposure categories are: CSE, cigarette smoke extract; IQOS, IQOS™ vapor extract; GLO, glo™ vapor extract; control, unexposed bacteria. Scale bar = 10 μm.

## Abbreviations

The following abbreviations are used in this manuscript:

ATCC	American Type Culture Collection
BHI	Brain Heart Infusion
CFU	Colony Forming Units
COPD	Chronic Obstructive Pulmonary Disease
ConA	Concanavalin A
CSC	Cigarette Smoke Condensate
CSE	Cigarette Smoke Extract
CV	Crystal Violet
DMSO	Dimethyl Sulfoxide
ECVE	Electronic Cigarette Vapor Extract
EPS	Extracellular Polysaccharides
FDR	False Discovery Rate
HPHC	Harmful and Potentially Harmful Constituent
HTP	Heated Tobacco Product
KW	Kruskal–Wallis
MIC	Minimum Inhibitory Concentration
MBC	Minimum Bactericidal Concentration
MTT	3-(4,5-Dimethylthiazol-2-yl)-2,5-diphenyltetrazolium bromide
NAD	Nicotinamide Adenine Dinucleotide
OD	Optical Density
PBS	Phosphate-Buffered Saline
PMMA	Polymethyl Methacrylate
RPM	Rotations Per Minute
sBHI	Supplemented Brain Heart Infusion (with hemin and β-NAD)
TSB	Tryptone Soy Broth
WGA	Wheat Germ Agglutinin
WHO	World Health Organization
HI	*Haemophilus influenzae*
KP	*Klebsiella pneumoniae*
PSD	*Pseudomonas aeruginosa*
SA	*Staphylococcus aureus*
SP	*Streptococcus pneumoniae*
