# Supplementary material for: Comparative Effects of Cigarette Smoke and Heated Tobacco Product Aerosols on Biofilm Production by Respiratory Pathogens
Source: Microorganisms. 2025 Oct 28;13(11):2459. doi: 10.3390/microorganisms13112459 (PMC12654149; doi:10.3390/microorganisms13112459)
Supplement: Supplementary file 1 [file microorganisms-13-02459-s001.zip › microorganisms-3931276-supplementary.pdf]

microorganisms

Supplementary File to "Comparative Effects of Cigarette Smoke and Heated Tobacco Product Aerosols on Biofilm Production by Respiratory Pathogens"

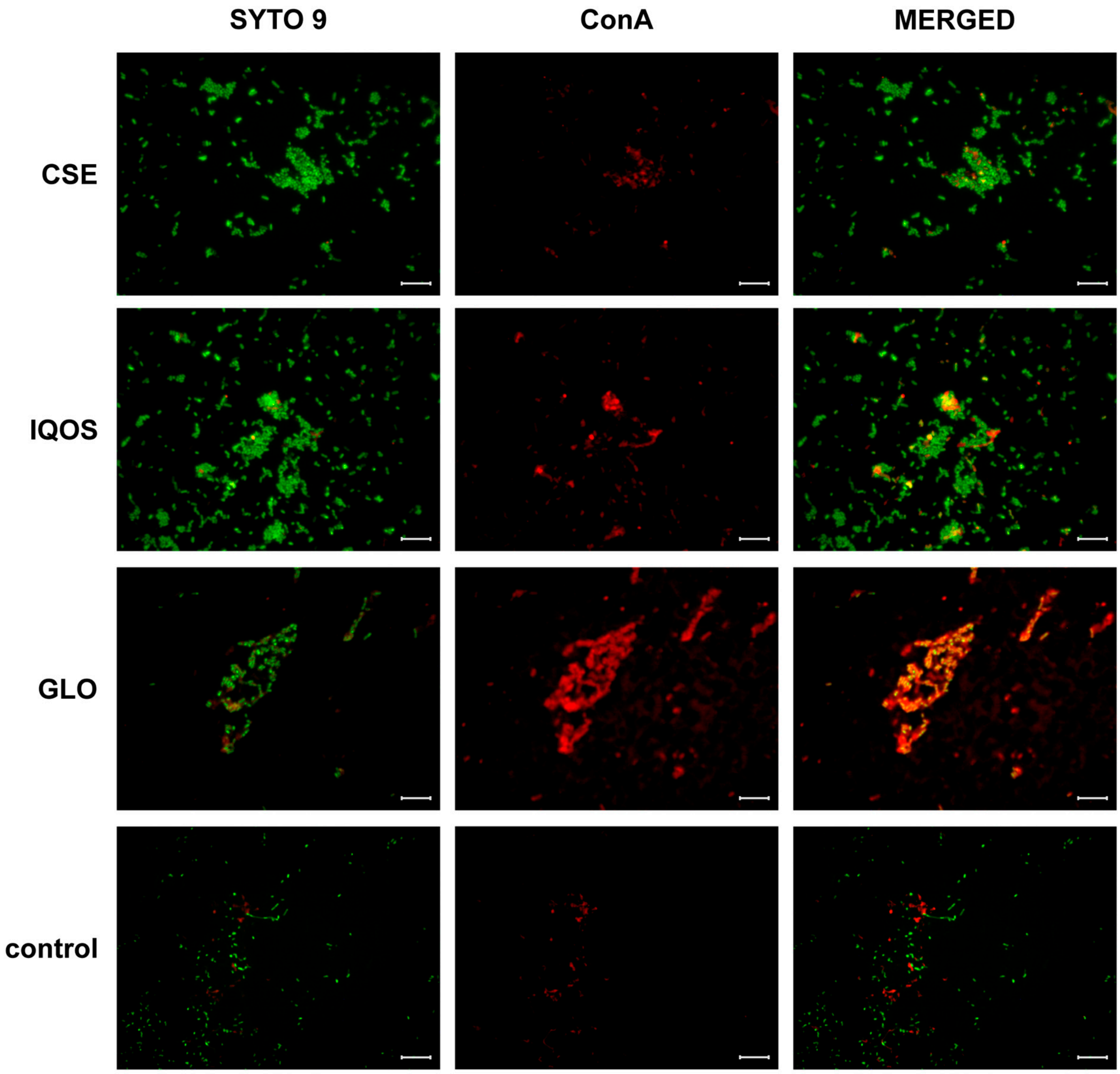

Figure S1. Epifluorescence microscopy images of 24-h *K. pneumoniae* biofilms. Nucleic acids were stained with SYTO® 9 (green), and polysaccharides were stained with concanavalin A conjugated to Texas Red® (ConA, red). Exposure categories are: CSE, cigarette smoke extract; IQOS, IQOS™ vapor extract; GLO, glo™ vapor extract; control, unexposed bacteria. Scale bar = 10 μm.

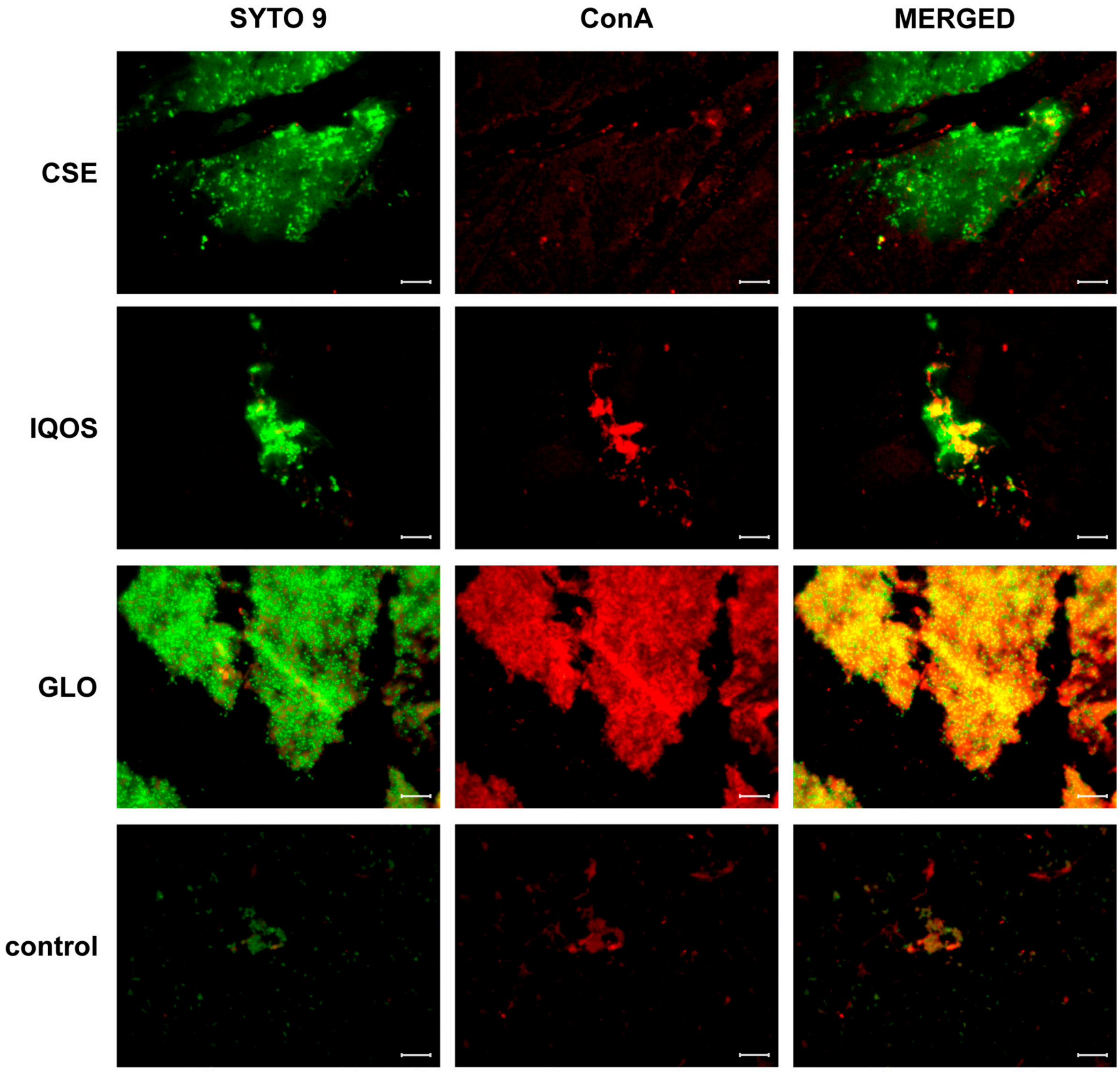

Figure S2. Epifluorescence microscopy images of 24-h *P. aeruginosa* biofilms. Nucleic acids were stained with SYTO® 9 (green), and polysaccharides were stained with concanavalin A conjugated to Texas Red® (ConA, red). Exposure categories are: CSE, cigarette smoke extract; IQOS, IQOS™ vapor extract; GLO, glo™ vapor extract; control, unexposed bacteria. Scale bar = 10 µm.

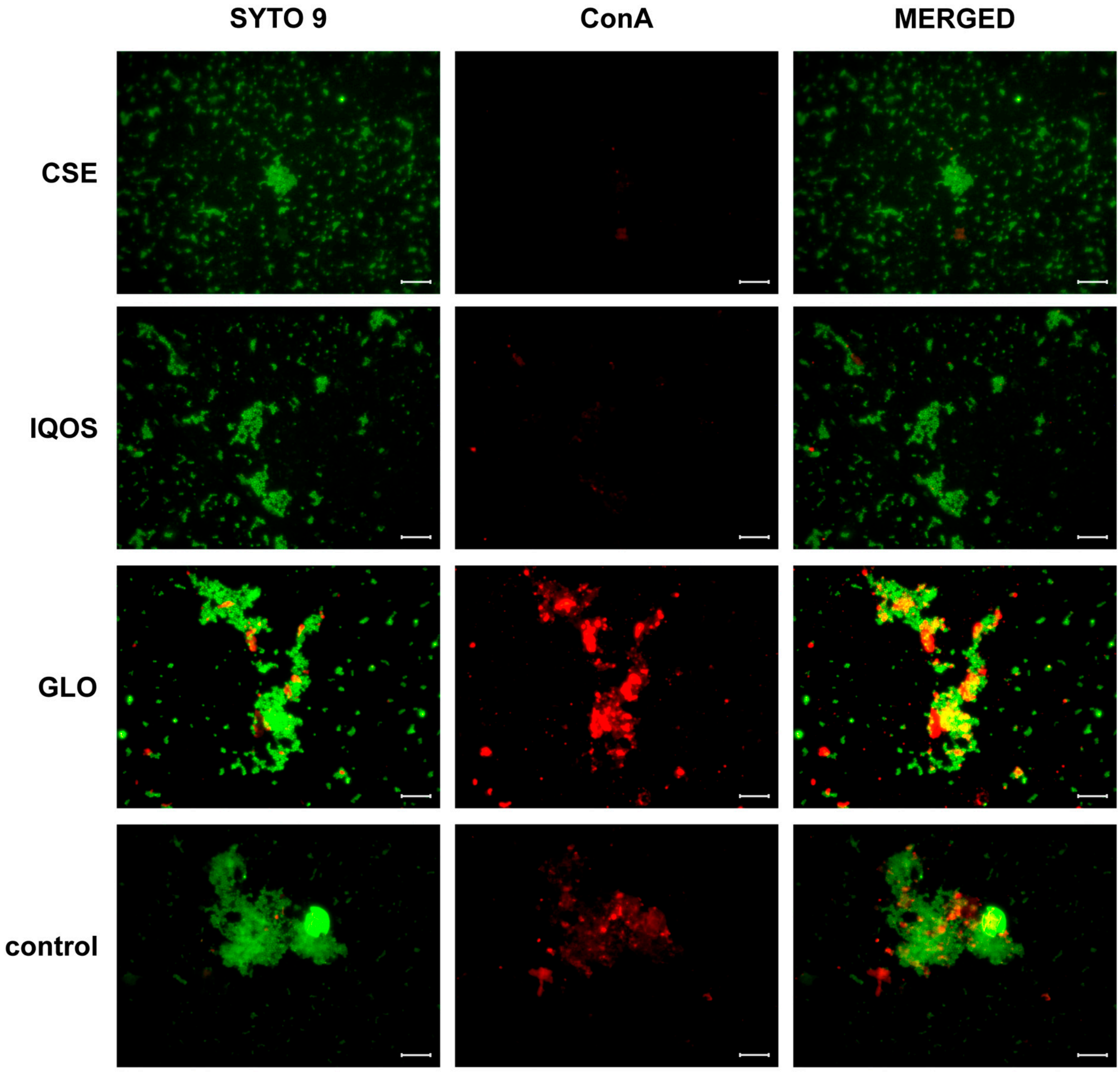

Figure S3. Epifluorescence microscopy images of 24-h *S. pneumoniae* biofilms. Nucleic acids were stained with SYTO® 9 (green), and polysaccharides were stained with concanavalin A conjugated to Texas Red® (ConA, red). Exposure categories are: CSE, cigarette smoke extract; IQOS, IQOS™ vapor extract; GLO, glo™ vapor extract; control, unexposed bacteria. Scale bar = 10 μm.
